# Correction to “Male Sex Is Associated With Severe Pulmonary Hypertension and Worse Outcome in Chronic Obstructive Pulmonary Disease Patients Referred for Right Heart Catheterization”

**DOI:** 10.1002/pul2.70338

**Published:** 2026-06-15

S. S. Shah, A. U. Rehman, S. Amin, M. Nawaz, and A. Laiba, “Male Sex Is Associated With Severe Pulmonary Hypertension and Worse Outcome in Chronic Obstructive Pulmonary Disease Patients Referred for Right Heart Catheterization,” *Pulmonary Circulation* 16 (2026): e70317, https://doi.org/10.1002/pul2.70317.

Due to a publisher's error, the article's title was incomplete in the published version. The correct title of the article is: “Letter to the Editor: Male Sex Is Associated With Severe Pulmonary Hypertension and Worse Outcome in Chronic Obstructive Pulmonary Disease Patients Referred for Right Heart Catheterization.”

We apologize for this error.

